# The Cellular Bromodomain Protein Brd4 has Multiple Functions in E2-Mediated Papillomavirus Transcription Activation

**DOI:** 10.3390/v6083228

**Published:** 2014-08-20

**Authors:** Christine M. Helfer, Junpeng Yan, Jianxin You

**Affiliations:** Department of Microbiology, University of Pennsylvania, Perelman School of Medicine, Philadelphia, PA 19104, USA; E-Mails: chelfer@mail.med.upenn.edu (C.M.H.); yanjunpeng1980@hotmail.com (J.Y.)

**Keywords:** papillomavirus, E2, Brd4, P-TEFb, JQ1, transcription

## Abstract

The cellular bromodomain protein Brd4 functions in multiple processes of the papillomavirus life cycle, including viral replication, genome maintenance, and gene transcription through its interaction with the viral protein, E2. However, the mechanisms by which E2 and Brd4 activate viral transcription are still not completely understood. In this study, we show that recruitment of positive transcription elongation factor b (P-TEFb), a functional interaction partner of Brd4 in transcription activation, is important for E2’s transcription activation activity. Furthermore, chromatin immunoprecipitation (ChIP) analyses demonstrate that P-TEFb is recruited to the actual papillomavirus episomes. We also show that E2’s interaction with cellular chromatin through Brd4 correlates with its papillomavirus transcription activation function since JQ1(+), a bromodomain inhibitor that efficiently dissociates E2-Brd4 complexes from chromatin, potently reduces papillomavirus transcription. Our study identifies a specific function of Brd4 in papillomavirus gene transcription and highlights the potential use of bromodomain inhibitors as a method to disrupt the human papillomavirus (HPV) life cycle.

## 1. Introduction

Human papillomavirus (HPV) is a common sexually transmitted pathogen associated with cervical and anal cancers, as well as certain cancers of the head and neck [1]. HPV-associated cancers arise after a prolonged persistent infection. While the HPV vaccines effectively protect against new HPV infections, they are ineffective at treating ongoing infections [2,3]. There are currently a small number of drugs that show promise as anti-viral treatments against HPV infections, but these drugs do not specifically target papillomaviruses and, therefore, cause many side effects [4]. For this reason, there is still a need for effective anti-viral treatments that more specifically target the HPV infection.

Papillomaviruses are small, double-stranded DNA viruses with an approximately 8 kb episomal genome that can be divided into three regions: the long control region (LCR), the early gene region, and the late gene region [5]. The LCR contains the origin of replication and a transcriptional enhancer region containing multiple binding sites for the viral E2 protein and cellular transcription factors [6]. There are six early viral genes: E1, E2, E4, E5, E6, and E7. These early genes are expressed from one or more early promoters as polycistronic mRNAs [5]. The prototypical papillomavirus, bovine papillomavirus 1 (BPV1), has six early promoters that each encode a different polycistronic transcript while the high-risk HPVs, such as HPV types 16 and 18, only contain one early promoter [7,8]. The polycistronic transcripts encoded from the early promoters utilize the same poly-adenylation site and are processed by cellular splicing factors [9].

Papillomavirus gene expression is strictly regulated by the differentiation status of the infected cell [10,11]. During initial infection in basal epithelial cells, small amounts of E1 and E2 are expressed from the early promoter to support low-level maintenance replication of the viral genome. The viral oncoproteins E6 and E7 promote proliferation of infected cells and establish a cellular environment conducive for the genome amplification stage of the papillomavirus life cycle [12]. Upon differentiation of the infected cells, the differentiation-dependent late promoter located within the E7 gene activates robust E1 and E2 expression to support viral genome amplification as well as expression of the L1 and L2 capsid proteins for assembly of new virions [10,11].

E2 serves as the master regulatory protein of viral early gene transcription by binding to several sites upstream of the early promoter to tightly regulate viral gene expression [13]. This tight regulation ensures that just enough E6 and E7 are expressed to drive differentiated cells into S phase while avoiding the development of neoplasias and carcinomas which can be induced by E6/E7 over-expression [12]. E2 can both activate and repress transcription from the early promoter and this is thought to be partially regulated by E2 levels where low levels of E2 activates viral transcription while elevated E2 levels repress the viral early promoter [14,15,16]. E2 also recruits cellular transcription factors and chromatin modulatory proteins to the early promoter to mediate viral transcription activation and repression [17,18,19,20,21,22].

Brd4 is an important factor for cellular transcription as well as transcriptional regulation of a variety of different viruses including papillomaviruses, Epstein-Barr virus, and human immunodeficiency virus (HIV) [23,24,25,26]. In cells, Brd4 interacts with the Cdk9 and Cyclin T1 subunits of positive transcription elongation factor b (P-TEFb), displacing the negative regulators, HEXIM1 and 7SKsnRNA, from the P-TEFb complex to transform P-TEFb into its transcriptionally active form [23,24,27]. Brd4 then recruits active P-TEFb to the transcription pre-initiation complex of many cellular genes where it stimulates transcription elongation by phosphorylating serine 2 in the *C*-terminal domain (CTD) of RNA polymerase II [24,26].

Interestingly, for papillomaviruses, Brd4 binding is important for E2’s dual transcription regulatory functions [21,22,28]. In E2-dependent luciferase reporter assays where E2 expression stimulates luciferase gene transcription, Brd4 knock-down or abrogation of the E2-Brd4 interaction inhibits E2 transactivation of the luciferase reporter [21,29,30]. Similarly, in luciferase reporter assays where E2 functions to repress luciferase gene expression, both Brd4 knock-down and disruption of the E2-Brd4 interaction reduce E2’s ability to repress expression of the reporter gene [22,28]. Furthermore, in cervical cancer cells where the HPV genome is integrated into the cellular DNA and the E2 gene is disrupted, Brd4 activates viral oncogene transcription independently of E2 by recruiting P-TEFb to the HPV early promoter. However, upon reintroduction of E2 into these cells, E2 functions to repress viral oncogene expression by interacting with Brd4 and competitively inhibiting the Brd4-P-TEFb interaction [31]. Wu* et al.* showed that the transcription repression function of E2 and Brd4 is at least partially mediated by preventing the assembly of the pre-initiation complex near the viral promoter [28]. In contrast, the exact mechanism(s) whereby Brd4 contributes to viral transcription activation during papillomavirus infection is still unknown.

In this study, we sought to uncover the mechanism underlying Brd4’s role(s) in E2-dependent transcription activation. Using an E2-responsive reporter assay, we demonstrate that Brd4 recruitment of P-TEFb is important for E2-dependent transactivation. We also found that P-TEFb is recruited to the papillomavirus genome. Furthermore, we provide evidence that Brd4 tethering of E2 to the cellular chromatin is necessary for the transactivation of the E2-responsive reporter. Lastly, in cells carrying the papillomavirus genomes, we demonstrate that inhibiting Brd4’s association with cellular chromatin using the bromodomain inhibitor, JQ1(+), effectively reduces transcription of the viral early genes E1, E2, E6, and E7. Together, these findings suggest that the Brd4 interaction with E2 is not only necessary for recruiting P-TEFb to the papillomavirus early promoter, but might also be important for tethering E2 and the viral genome complexes to particular regions of the cellular chromatin to support viral gene transcription.

## 2. Results 

### 2.1. P-TEFb Is Important for Papillomavirus E2-Mediated Transcription Activation

**Figure 1:**
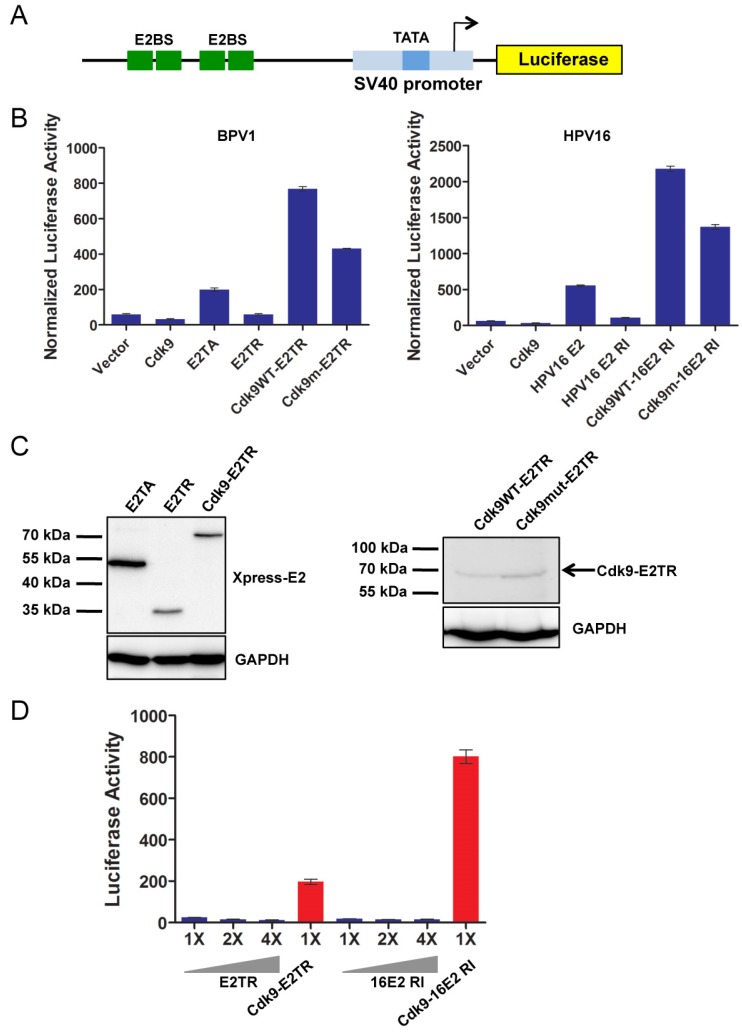
P-TEFb is important for E2-mediated papillomavirus transcription activation. (**A**) Schematic diagram of the 2x2xE2BS-Luciferase reporter. Two pairs of E2 binding sites (E2BS) are upstream of the minimal SV-40 promoter and firefly luciferase gene; (**B**) C33A cells were cotransfected with p2x2xE2BS-Luciferse, CMV-β-gal, and either an empty vector (Vector), a Cdk9 expression plasmid (Cdk9), or the indicated E2 expression plasmids. Cells transfected with BPV1 E2TA expression constructs are presented in the left panel while cells transfected with HPV16 E2 expression constructs are presented in the right panel. Forty-eight hours post transfection, the cells were processed for luciferase and β-galactosidase measurements. The luciferase values were normalized to β-galactosidase expressed from a constitutive CMV promoter. Average and standard deviation were calculated from three experiments; (**C**) Nuclear proteins from cells transfected as in (**B**) were extracted and immunoblotted using anti-Xpress and anti-GAPDH antibodies (see also Figure S3); (**D**) Cells were cotransfected as in (**B**) but with 1× of the Cdk9-E2 fusion constructs or increasing amounts of E2TR/16E2 RI (1×, 2×, or 4×). Empty vector was used to make the amount of DNA used for each transfection equal. Forty-eight hours post transfection, the cells were processed for luciferase activity and β-galactosidase activity measurements. Average and standard deviation were calculated from three experiments.

Brd4 has previously been shown to assist E2 in transactivating viral genes but Brd4’s precise role in this process has remained elusive [21,29]. As Brd4 functions in cellular transcription activation mainly by recruiting P-TEFb to gene promoters, we decided to investigate whether Brd4 activates papillomavirus transcription by recruiting P-TEFb to the viral promoters. We first utilized an E2-responsive luciferase reporter assay to study this E2 transactivation function [21,32]. The p2x2xE2BS-luciferase reporter construct contains two pairs of E2 binding sites upstream of a minimal SV40 promoter and the luciferase gene (Figure 1A). When either BPV1 E2TA or HPV16 E2 are cotransfected with the reporter plasmid, it binds the E2 binding sites and activates the expression of luciferase (Figure 1B). As previously reported, however, the E2 mutants, BPV1 E2TR and HPV16 E2 R37A/I73A (16E2 RI), which do not bind Brd4, are unable to transactivate the luciferase reporter (Figure 1B) [21,29]. We next determined if the defect in transcription activation observed in the Brd4 binding-deficient E2 mutants is due to their inability to recruit P-TEFb through Brd4 association. To test this, we fused the Cdk9 subunit of P-TEFb to the E2 mutants, E2TR or 16E2 RI, and tested them in the luciferase reporter assay. As shown in Figure 1B, Cdk9 fusion with either E2TR or 16E2 RI mutant markedly restored the transactivation activities well above that seen for wild type E2TA and 16E2. This enhanced transactivation activity was also observed when Cdk9 was fused to 16E2 WT or E2TA [33]. The Cdk9 fusion to E2TR expressed at similar level as E2TR protein (Figure 1C) but the HPV16 E2 proteins express below the level detectable by Western blot, thus, it was hard to compare their protein levels. However, because both E2TR and 16E2 RI have been shown by us and others to be completely inactive in the E2 transactivation reaction [29,34], this highly stimulated transactivation activity observed with the Cdk9-E2TR and Cdk9-16E2 RI constructs was not likely due to higher expression of these fusion proteins relative to E2TR or 16E2 RI. We further proved this by comparing the transactivation activity of low levels (1×) of Cdk9-E2TR or Cdk9-16E2 RI with 1×, 2×, and 4× more of E2TR or 16E2 RI in the luciferase reporter assay. As shown in Figure 1D, while E2TR and 16E2 RI could not transactivate the luciferase reporter even when four times more expression construct was transfected, very low levels of the Cdk9-E2 mutant fusion proteins still robustly transactivated the luciferase reporter, confirming that the restored transactivation by the Cdk9-E2 mutant fusion proteins is not likely due to increased protein levels. It rather suggested a gain of function introduced by the Cdk9 moiety of the fusion proteins. These results demonstrate that P-TEFb is important for E2 transactivation function, indicating that Brd4 likely functions to recruit P-TEFb to the E2-responsive promoter to support viral transcription.

We next tested whether fusing the E2 mutants to a kinase-dead Cdk9 D167N mutant (Cdk9m) abolished the transactivation activity of the fusion proteins [35]. The D167N mutation did not affect the Cdk9-E2 fusion protein levels (Figure 1C). These mutant Cdk9-E2 fusion constructs transactivated the luciferase reporter expression at a consistently lower level than the E2 mutants fused to wild-type Cdk9, although their activity was still well above that seen for WT E2 proteins (Figure 1B). This is likely because the Cdk9 D167N mutant retains some low-level kinase activity. Alternatively, because this Cdk9 D167N mutant can still bind Brd4, and we have previously shown that Brd4 can form Brd4-Brd4 homodimers [26,36], we suspect that Brd4 dimers bound to Cdk9 D167N-E2 can recruit endogenous P-TEFb to the luciferase reporter to partially activate transcription elongation of the luciferase gene.

### 2.2. P-TEFb Is Recruited to the Papillomavirus Genome

**Figure 2:**
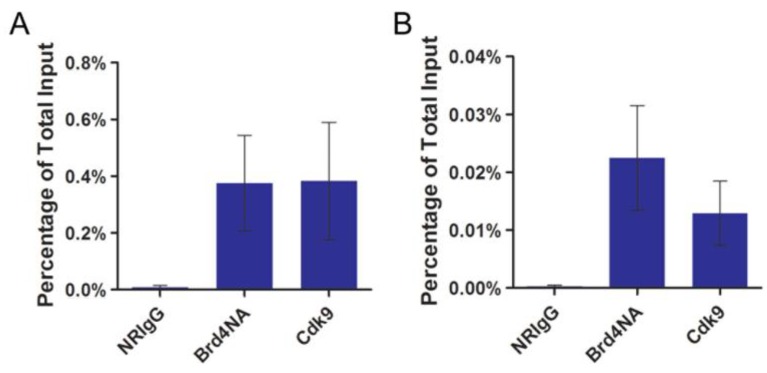
P-TEFb is recruited to the papillomavirus genome. (**A**) H2 cells were subjected to ChIP assay using normal rabbit IgG (NRIgG), Brd4NA antibody, or anti-Cdk9 antibody. ChIP samples were analyzed by qPCR using primers targeting the BPV1 genome; (**B**) W12 (clone 20863) cells were subjected to ChIP assay as in (**A**) and ChIP samples were analyzed by qPCR using primers targeting the HPV16 genome. For both (**A**) and (**B**), the values represent the average and standard deviation of three independent experiments.

To confirm the role of P-TEFb in viral genome transcription, we tested if P-TEFb is recruited to the native papillomavirus genome. For this set of experiments we used the H2 and W12 (clone 20863) cells that maintain either BPV1 or HPV16 episomal genomes, respectively [37,38]. Both of these cell types stably maintain the viral episomes and support papillomavirus early promoter transcription [39,40,41,42]. ChIP assays were performed with a Cdk9 antibody to examine the occupancy of P-TEFb on the papillomavirus genome. As a positive control, an affinity purified Brd4 *N*-terminal antibody was also used in the ChIP analysis since Brd4 is known to associate with the papillomavirus genome through its interaction with E2 [34]. Primers recognizing either the BPV1 or HPV16 genome were used in ChIP quantitative PCR (qPCR) to detect the binding of P-TEFb and Brd4 to the viral episome as we have described previously [42]. Both the Cdk9 and Brd4 antibodies immunoprecipitated the BPV1 and HPV16 genomes at significantly higher levels than the negative control ChIP using normal rabbit IgG (NRIgG) (Figure 2A,B). Importantly, no BPV1 or HPV16 signal was detected by qPCR from ChIP experiments using uninfected C127 cells, confirming that these primers specifically amplify a segment of the viral genome [33]. These results demonstrate that P-TEFb is recruited to the papillomavirus episome likely through interaction with Brd4.

### 2.3. Brd4-CTD Disrupts Papillomavirus Transcription Activation by the Cdk9-E2 Fusion Proteins

**Figure 3:**
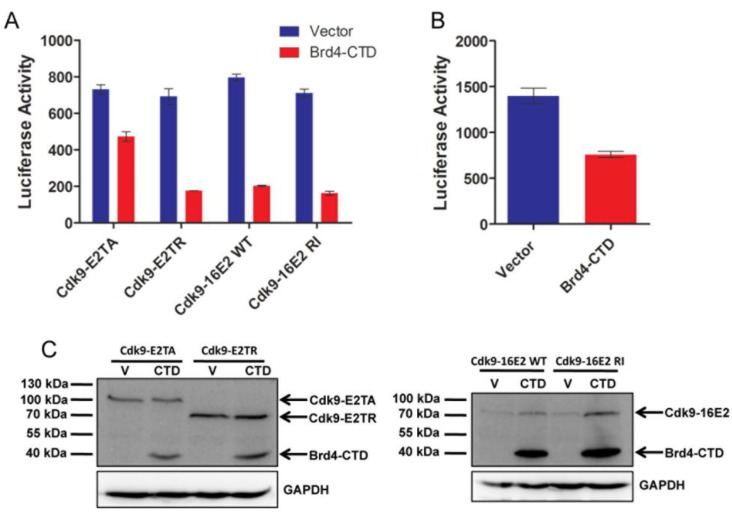
Brd4-CTD inhibits transcription activation by the Cdk9-E2 fusion proteins. (**A**) Along with the p2x2xE2BS-Luciferase construct, C33A cells were cotransfected with the indicated Cdk9-E2 fusion constructs and either an empty vector (Vector) or Brd4-CTD expression construct at a 1:6 ratio (Cdk9-E2:V/CTD). Forty-eight hours post transfection, the cells were processed for luciferase activity measurement; (**B**) Cells were transfected as in (**A**) except a 1:8 ratio of Cdk9-E2TA:V/CTD was used. Forty-eight hours post transfection, the cells were processed for luciferase activity measurement. In (**A**) and (**B**), average and standard deviation were calculated from three experiments; (**C**) Cells were transfected as in (**A**) and nuclear lysates were immunoblotted with anti-Xpress and anti-GAPDH antibodies (see also Figure S4).

E2 and P-TEFb both interact with the extreme *C*-terminus of Brd4 and expression of the Brd4-CTD effectively inhibits Brd4’s interaction with these two proteins [24,26,31,34]. In addition, Brd4-CTD abrogates E2 transactivation of the p2x2xE2BS-luciferase reporter [21]. Our findings suggest that this is likely because Brd4-CTD prevents the recruitment of Brd4 as well as P-TEFb to the E2-responsive promoter. We hypothesized that if Brd4’s only function in E2 transactivation is to recruit P-TEFb, then inhibiting Brd4’s interaction with E2 and P-TEFb using Brd4-CTD will likely have no effect on transactivation function of the E2 proteins that are already fused to P-TEFb. Surprisingly, when Cdk9-E2 fusion constructs were coexpressed with the Brd4-CTD expression construct, Brd4-CTD significantly inhibited the transactivation function of all Cdk9-E2 fusions (Figure 3A). The weaker inhibition of Cdk9-E2TA by Brd4-CTD is likely because E2TA has a stronger binding affinity to Brd4 than HPV16 E2, thus, more Brd4-CTD is needed to efficiently break the E2TA-Brd4 interaction [29]. Indeed, we found that higher concentration of Brd4-CTD dramatically inhibited transactivation by Cdk9-E2TA (Figure 3B). For an unknown reason, Brd4-CTD consistently increased β-galactosidase expression from the CMV promoter, which prevented us from normalizing the luciferase activity to β-galactosidase activity. We, therefore, excluded the β-galactosidase expression construct from later transfection reactions and instead used E2 protein levels as controls for transfection efficiency. Western blot analysis showed that Brd4-CTD expression does not affect the levels of Cdk9-E2TA or Cdk9-E2TR levels. Instead, as previously reported, Brd4-CTD even caused moderate stabilization of Cdk9-16E2 WT and Cdk9-16E2 RI proteins (Figure 3C) [43]. As the P-TEFb complex is already tethered to E2 by direct fusion, these results suggested that, other than recruiting P-TEFb, Brd4 probably has additional roles in E2-mediated transactivation. Alternatively, it is possible that Brd4-CTD binds Cdk9 in the E2 fusions and induces a conformational change that abrogates P-TEFb function.

### 2.4. Releasing Brd4 from Chromatin by JQ1(+) Correlates with Diminished E2-Mediated Transcription Activation

**Figure 4:**
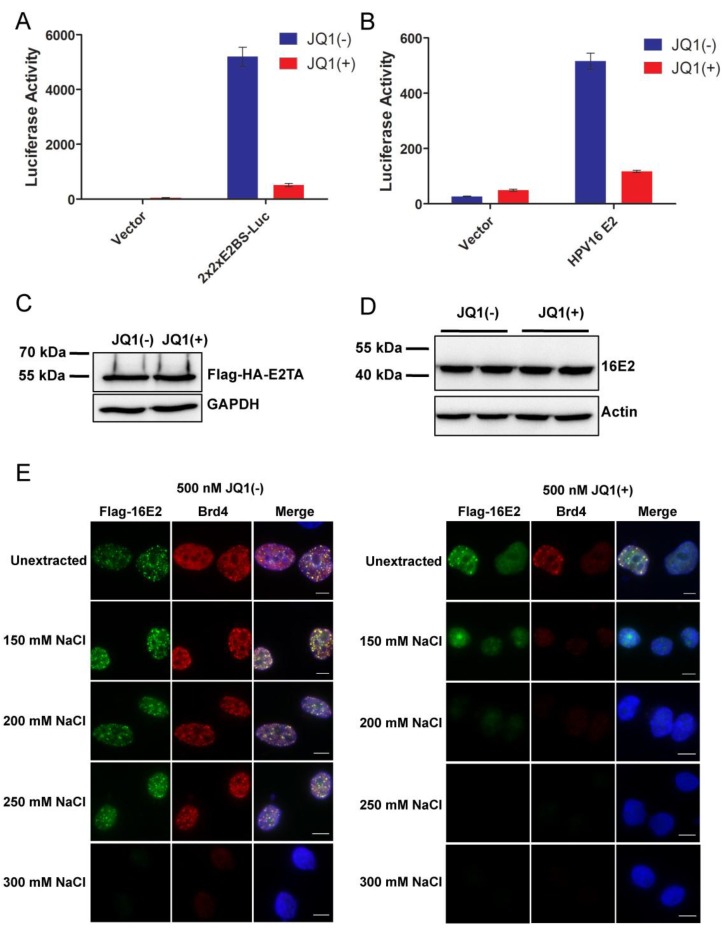
JQ1(+) releases E2 from chromatin and inhibits E2 transcription activation. (**A**) C33A cells stably expressing Flag-HA-tagged BPV1 E2TA were transfected with either empty vector (Vector) or the p2x2xE2BS-Luciferase construct. At thirty-three hours post transfection, the cells were treated with 500 nM JQ1(−) or JQ1(+) until collection at forty-eight hours post transfection when the cells were processed for luciferase activity measurement; (**B**) C33A cells were cotransfected with the p2x2xE2BS-Luciferase construct and either an empty vector (Vector) or the Flag-16E2 construct. As in (**A**), the cells were treated with 500 nM JQ1(−) or JQ1(+) for 15 h then collected at forty-eight hours post transfected for luciferase activity measurement. Average and standard deviation were calculated from three experiments for (**A**) and (**B**); (**C**) Cells stably expressing Flag-HA-tagged BPV1 E2TA were transfected and treated with JQ1 as in (**A**). Nuclear proteins were harvested and immunoprecipitated with anti-Flag (M2) beads. The precipitates were immunoblotted with anti-HA-HRP antibody. GAPDH levels were analyzed from the input with anti-GAPDH antibody (see also Figure S5); (**D**) C33A cells were transfected and treated with JQ1 as in (**B**). Protein extracts were immunoblotted with anti-HPV16 E2 and anti-actin antibodies; (**E**) C33A cells were cotransfected with Flag-16E2 and Brd4 expression constructs at a 1:1 ratio. Two hours prior to collection, cells were treated with 500 nM JQ1(−) or JQ1(+). Forty-eight hours post transfection, coverslips were collected and either fixed immediately (unextracted) or pre-extracted in buffer containing the indicated concentration of NaCl prior to fixation. The coverslips were then stained with anti-Flag (green) and anti-Brd4CA (red) antibodies and counterstained with DAPI. This experiment was repeated a total of three times and the most representative images are presented. Bar, 5 μm.

Previous studies published by Jang* et al.* showed that Brd4 directs E2 to transcriptionally active regions of cellular chromatin [44,45]. We hypothesized that this recruitment of E2 by Brd4 to active regions of the nucleus would give the virus easy access to the cellular transcription machinery and thereby assist in E2-mediated transcription activation. In this case, inhibiting Brd4 chromatin binding would likely impair E2-mediated transactivation. To examine the importance of Brd4’s association with chromatin for E2 transactivation function, we abrogated Brd4’s interaction with acetylated histones on chromatin using a small molecule inhibitor, JQ1(+), as we have described previously [46,47]. JQ1 is a novel thieno-triazolo-1,4-diazepine that competitively binds to acetyl-lysine recognition motifs of bromodomains with highest specificity and potency toward the BET family of proteins [48]. Therefore, it is an effective small-molecule to inhibit Brd4 interaction with chromatin [48]. C33A cells stably expressing BPV1 E2TA were transfected with the p2x2xE2BS-Luciferase construct or an empty vector and then treated with 500 nM of JQ1(+) or its inactive stereoisomer, JQ1(−), for 15 h prior to the luciferase assay. As shown in Figure 4A, E2TA transactivation of the luciferase reporter was drastically reduced in cells treated with JQ1(+) while cells transfected with empty vector had very low background luciferase activity. A similar result was observed when C33A cells were transfected with the HPV16 E2 and p2x2xE2BS-Luciferase constructs and treated for 15 h with 500 nM of JQ1(+) or JQ1(−) prior to the luciferase assay (Figure 4B). Similar to Brd4-CTD expression, JQ1(+) consistently increased β-galactosidase levels precluding us from normalizing the luciferase reporter to this protein so the β-galactosidase construct was excluded from the experiments presented in Figure 4. The E2 protein levels, however, were unaffected by JQ1(+) treatment (Figure 4C,D). Moreover, low level of HPV16 E2 could still robustly activate luciferase expression in the presence of JQ1(−), whereas high level of E2 protein was transcriptionally inactive with JQ1(+) treatment (Figure S1). This reduction in luciferase expression was also observed with as low as 100 nM JQ1(+) and as short as 8 h of treatment [33]. These results suggested that Brd4 binding to chromatin is essential for E2 transactivation activity. Together with the findings by Jang* et al.*, we hypothesized that chromatin-bound Brd4 functions to direct E2 to transcriptionally active regions of the nucleus to facilitate E2 transcription activation [44,45].

To confirm that JQ1(+) abolishes E2’s association with cellular chromatin through interaction with Brd4, the strength of E2’s chromatin association in the presence of JQ1(−) or JQ1(+) was examined as described by McPhillips* et al.* [49]. When HPV16 E2 and Brd4 are ectopically expressed in C33A cells, the E2-Brd4 complexes bound to cellular chromatin appear as nuclear speckles in immunofluorescently-stained cells (Figure 4E and [46]). Treatment with JQ1(−) did not affect the localization of E2 and Brd4 in these speckles (Figure 4E, JQ1(−) unextracted). However, after only 2 h of JQ1(+) treatment, E2 and Brd4 no longer localized to small speckles but instead formed larger, punctate nuclear spheres or became diffuse in the nucleus (Figure 4E, JQ1(+) unextracted). These large spheres formed by E2 and Brd4 in the presence of JQ1(+) have been described previously and were shown to be E2-Brd4 complexes that form off the cellular chromatin [46]. The strength of E2’s binding to cellular chromatin was next tested by pre-extracting proteins using solutions with increasing salt concentrations prior to cell fixation. In this experiment, the cells were incubated in buffer containing NP-40 to permeablize the nucleus and in different salt concentrations ­to extract proteins unbound or weakly bound to cellular chromatin. All proteins besides those tightly bound to chromatin were washed away and then the cells were fixed for immunofluorescence analysis. Upon JQ1(−) treatment, Flag-16E2 and Brd4 remained associated with cellular chromatin in nuclear speckles after pre-extraction with buffer containing up to 250 mM NaCl (Figure 4E). Conversely, with JQ1(+) treatment and pre-extraction with 150 mM NaCl, only diffuse Flag-16E2 and Brd4 staining was detected in the nucleus but pre-extraction with higher salt concentrations removed nearly all of the Flag-16E2 and Brd4 signals (Figure 4E). Similar results were observed with BPV1 E2TA [33]. This experiment demonstrates that Brd4 release from chromatin by JQ1(+) treatment weakens E2’s association with chromatin, confirming that Brd4 mediates E2 binding to cellular chromatin.

### 2.5. JQ1(+) Treatment Reduces Papillomavirus Gene Expression

**Figure 5:**
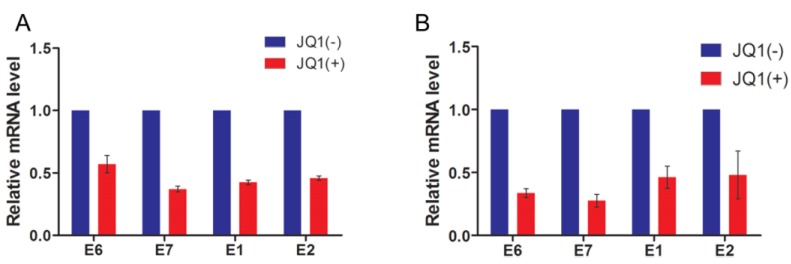
JQ1(+) treatment inhibits papillomavirus early gene expression. (**A**) H2 cells were treated with 1 μM JQ1(–) or JQ1(+) for 15 h. RNA from these cells was reverse transcribed and the levels of the indicated BPV1 early gene mRNAs were measured by RT-qPCR. The mRNA level for each viral gene was normalized to GAPDH mRNA level. The JQ1(+)-treated sample values are presented relative to the JQ1(–) sample values; (**B**) W12 (clone 20863) cells were treated with 100 nM JQ1(–) or JQ1(+) for 15 h. As in (**A**), RNA was collected and reverse transcribed and the cDNA was used in RT-qPCR analyses to measure HPV16 early gene mRNAs. The mRNA level for each viral gene was normalized to GAPDH mRNA level. The JQ1(+)-treated sample values are presented relative to the JQ1(–) sample values. For (**A**) and (**B**), average and standard deviation were calculated from three independent experiments.

In the next experiment, we examined whether JQ1(+)-induced dissociation of Brd4 from chromatin could also repress E2-mediated transactivation of gene transcription from the native papillomavirus genome. For this study, H2 and W12 (clone 20863) cells were treated with JQ1(–) or JQ1(+) for 15 h prior to mRNA isolation. Viral gene expression was measured via quantitative reverse transcription PCR (RT-qPCR) using primers specific for either the BPV1 or HPV16 early genes: E1, E2, E6, and E7. As shown in Figure 5, JQ1(+) treatment caused efficient reduction in expression of all the viral early genes compared to JQ1(–) treatments. This reduction in viral transcripts was not due to a drop in papillomavirus episome levels from the JQ1(+) treatment (Figure S2). Notably, GAPDH mRNA levels were not significantly affected by the JQ1(+) treatment, indicating that JQ1(+) did not induce a global shutdown of cellular gene transcription. It is also important to note that both H2 and W12 cells looked healthy and grew normally after 15 h of JQ1(+) treatment, suggesting that the reduced viral mRNA levels did not result from cellular toxicity caused by JQ1(+). These results, together with the findings in Figure 4, suggest that JQ1(+) releases E2 and Brd4 from cellular chromatin and this dissociation correlates with dramatically reduced transcription of the papillomavirus early genes. However, the papillomavirus genome also associates with cellular histones and assembles into chromatin so it is possible that the Brd4 bromodomains directly interact with viral chromatin and abrogating this interaction might also inhibit viral transcription [50,51].

## 3. Discussion

**Figure 6:**
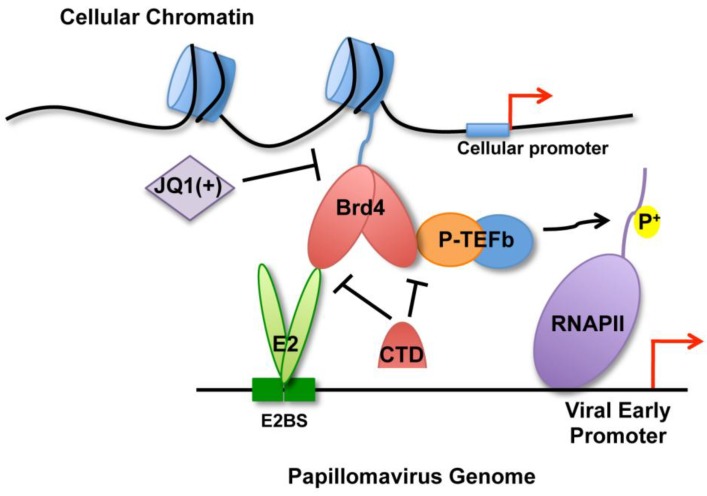
A model of Brd4 functions in E2-mediated viral transcription activation. Papillomavirus E2 protein binds to the E2 binding sites (E2BS) upstream of the early promoter as a dimer. E2 interacts with Brd4 at the *C*-terminus while the *N*-terminal Brd4 bromodomains direct E2 and the viral genome to transcriptionally active regions of the cellular genome. Brd4 can form homodimers and also recruit P-TEFb to the viral promoter to phosphorylate the RNA pol II CTD to activate transcription elongation. However, JQ1(+) blocks Brd4 association with cellular chromatin, impeding the recruitment of the viral genomes to transcriptionally active areas of the cellular genome and thereby inhibiting E2-mediated transcription activation of the papillomavirus promoters. The Brd4-CTD can abrogate the E2-Brd4 interaction as well as the Brd4-Cdk9 interaction, thus blocking P-TEFb recruitment to the viral promoter and preventing the activation of transcription elongation by P-TEFb.

Brd4 has long been known to function in papillomavirus transcription activation but its precise role in this process remained elusive. Here, P-TEFb was found recruited to the papillomavirus genome along with Brd4 and was also shown to restore transactivation function to Brd4 binding-deficient E2 mutants, suggesting an important role of Brd4/P-TEFb recruitment for papillomavirus transcription activation. Furthermore, our work is the first to identify JQ1(+) as a potent inhibitor of papillomavirus gene expression, suggesting an important role of Brd4’s chromatin association for viral transcription regulation. These findings support a model of Brd4 function in E2-dependent viral transcription activation (Figure 6).

E2 and Cdk9 both interact with the extreme *C*-terminal region of Brd4 [24,26,31,34]. This raises the question as to how Brd4 associates with E2 at the viral promoter while also recruiting P-TEFb. Our earlier report demonstrated that Brd4 proteins can interact intermolecularly to form homodimers [36]. We therefore predict that Brd4 dimerizes at the papillomavirus transcription complex (Figure 6). It has been well established that low levels of E2 activate viral transcription whereas elevated E2 levels repress the viral early promoter [14,15,16]. It is possible that, in the presence of low-level E2 expression, one Brd4 protein in the homodimers associates with E2 on the viral promoter while the other recruits P-TEFb. These molecular interactions therefore allow E2 to recruit the Brd4/P-TEFb complexes to the viral promoter to stimulate viral transcription activation (Figure 6). On the other hand, high levels of E2 proteins can saturate all of the Brd4 *C*-terminal binding sites, thus replacing the Brd4-P-TEFb interaction and preventing the recruitment of P-TEFb to the viral promoter region for transcription activation. This could eventually lead to repression of viral transcription as has been observed when E2 is ectopically expressed in cells with integrated HPV genomes [31]. Future studies will test this hypothesis and determine whether Brd4 dimerization is essential for PV transcription regulation.

JQ1(+) treatment potently inhibited transactivation of the p2x2xE2BS-Luciferase reporter and the papillomavirus early promoters. This transcription inhibitory effect by JQ1(+) is specific for papillomaviruses since GAPDH mRNA levels were not significantly affected by JQ1(+) and expression of β-galactosidase from a constitutive CMV promoter was actually elevated with JQ1(+) treatment. This observation also suggests that JQ1(+)-induced inhibition of E2 transcription activation is not a consequence of global gene expression shutdown. In fact, it was recently found that JQ1(+) effectively reactivates HIV transcription in latently infected cells [52,53]. The JQ1(+)-induced inhibition of E2 transcription activation was also observed with only 8 h of treatment, suggesting that this inhibition effect is not likely due to an impact on the cell cycle. Furthermore, we did not detect a significant change in papillomavirus genome levels in H2 and W12 cells after 15 h of JQ1(+) treatment. In a previous study we showed that JQ1(+) activates HPV16 genome replication in a transient transfection experiment, which would likely result in increased viral genome copies [47]. However, with the drastically reduced viral transcript levels in combination with abrogated viral genome maintenance in dividing cells infected with native viruses [46], we predict that prolonged JQ1(+) treatment would eventually induce a pronounced loss of the papillomavirus episomes.

Other than Brd4, JQ1(+) also affects the chromatin association of all BET family members as well as other bromodomain-containing proteins [48]. We, therefore, sought to specifically inhibit Brd4 chromatin binding to determine if the reduced papillomavirus transcription is exclusively from Brd4 inhibition. We previously utilized ectopic expression of the Brd4 double-bromodomains (BDI/II) to dissociate Brd4 from chromatin and showed that Brd4 BDI/II does not affect Brd2 chromatin binding, indicating that this is a more specific method to abrogate Brd4 chromatin interactions [36]. Unfortunately, although Brd4 BDI/II efficiently inhibited the E2 transactivation activities, it also drastically destabilized the E2 protein, preventing us from fairly assessing the effect of BDI/II on E2-mediated transcription activation [33]. However, because Brd4 is the only bromodomain-containing protein that carries the *C*-terminal domain for E2 binding, the observed JQ1(+) effects on E2 chromatin association and transcription activation are not likely resulted from the inhibition of other JQ1(+) targets besides Brd4.

Our analysis of E2 and Brd4 chromatin association upon JQ1(+) treatment demonstrates that E2’s chromatin association matches that of Brd4. Given that the other BET family members do not interact with E2, we believe this experiment suggests that Brd4 tethering of E2 to cellular chromatin might play an important role in E2 transcription activation of viral genes. Our observations are in line with the study published by Schweiger* et al.*, which showed that Brd4 binding is required for E2 transcriptional activation [21]. They found that inhibiting E2-Brd4 with Brd4-CTD or knocking-down Brd4 with Brd4-targeting siRNA significantly reduced E2-dependent transcription activation, demonstrating a role for Brd4 in the transcriptional activation function of E2. In studies from the McBride group, it was shown that Brd4 directs E2 to actively transcribed regions of cellular chromatin [44,45]. We predict the virus might rely on Brd4 to take its genome to areas of the nucleus where it can efficiently co-opt the cellular transcriptional machinery. However, this is not the only possible explanation to account for our findings so further studies are needed to confirm this hypothesis.

Brd4 is involved in transcription activation of a number of cellular genes and JQ1(+) has been shown to inhibit transcription of some of these genes [54,55,56,57]. It is therefore possible that at least part of the reduction in papillomavirus transcription from JQ1(+) treatment is due to a loss of cellular transcription factors important for papillomavirus transcription. Furthermore, Brd4 might contribute other functions necessary for viral transcription. For instance, the papillomavirus episomal genome assembles into chromatin with cellular histones and it is possible that Brd4 binds acetylated histones on the viral genome [50,51]. This binding might serve to recruit chromatin modulating factors or other transcription factors to the viral promoters. Additionally, we have observed that Brd4 can phosphorylate E2* in vitro* [58]. It will be interesting to determine if this Brd4 function can be detected in cells once a suitable antibody is generated and if this could be a regulatory switch by which Brd4 regulates E2’s transcription functions. Uncovering the mechanisms underlying E2 and Brd4 function in viral transcription regulation will provide a better understanding of how HPV gene expression is regulated throughout the viral life cycle.

We previously showed that JQ1(+) has the potential to be a valuable tool to eliminate HPV infection since it can not only release E2-Brd4 complexes from mitotic chromosomes, which could disrupt viral episome maintenance, but can also stimulate HPV16 DNA replication, which could trigger an early and inopportune anti-viral immune response [46,47]. In this study, we further discovered that JQ1(+) also abrogates papillomavirus gene expression. Future studies will address the important question of whether the combined effects of JQ1(+) on the HPV life cycle result in clearance of HPV persistent infection.

## 4. Materials and Methods

### 4.1. Cell Culture and Cell Lines

The HPV-negative cervical cancer cell line, C33A, and the mouse fibroblast cell line maintaining BPV1 episomes, H2, were maintained as monolayers in high glucose Dulbecco’s modified Eagle’s medium (DMEM) (Invitrogen, Carlsbad, CA, USA) containing 10% fetal bovine serum (FBS) (Hyclone, Logan, UT, USA) and 1% penicillin-streptomycin (Invitrogen). Generation of the C33A cells stably expressing Flag-HA-tagged E2TA was described previously [34]. The HPV16-positive cervical cancer epithelial cells, W12 (clone 20863), were provided by Dr. Paul Lambert and were maintained at subconfluence on mitomycin-c treated 3T3M feeder cells in F medium composed of 1 part DMEM and 3 parts F-12 medium (Life Technologies) supplemented with 5% FBS, 8.4 ng/mL cholera toxin (Sigma, St. Louis, MO, USA), 24 μg/mL adenine (Sigma), 10 ng/mL epidermal growth factor (R&D Systems, Minneapolis, MN, USA), 0.4 μg/mL hydrocortisone (Millipore, Billerica, MA, USA), and 5 μg/mL insulin (Sigma) [59].

### 4.2. Transient Transfections

For the luciferase reporter assays and Western blot analysis, cells were transfected using the calcium phosphate transfection method where 1.7 µg DNA was mixed with 10.1 µL 2 M CaCl_2_ in an 85 µL final volume. DNA/CaCl_2_ mixture was slowly dropped into 85 µL 2× HBS (55 mM HEPES, 0.4 M NaCl and 1.5 mM Na_2_HPO_4_, pH 7.0) while vortexing. The DNA mixture was then overlayed onto the cells with culture medium in a 6-well plate format. For immunofluorescence staining, cells were transfected with Lipofectamine 2000 (Invitrogen) following the manufacturer’s instructions.

### 4.3. Reagents

The Brd4 bromodomain-specific inhibitor, JQ1(+), and its inactive stereoisomer, JQ1(−), were provided by Dr. James Bradner and were dissolved in dimethyl sulfoxide (DMSO) and stored as 1000× stocks. The compound, 4',6-diamidino-2-phenylindole (DAPI), was dissolved in water as a 500× stock (Invitrogen). The primary antibodies used include: anti-Flag M2 (Sigma), anti-HA-HRP (Roche, Basel, Switzerland), anti-HPV16 E2 (Millipore), anti-Xpress (Invitrogen), anti-Cdk9 (sc-484) (Santa Cruz, Dallas, TX, USA), anti-GAPDH (US Biological, Salem, MA, USA), anti-Actin (Chemicon, Temecula, CA, USA), normal rabbit IgG (Millipore), anti-Brd4NA (recognizing Brd4 aa156-284), and anti-Brd4CA (recognizing Brd4 aa1313-1362). The secondary antibodies used for Western blotting were horseradish peroxidase (HRP)-linked anti-rabbit or anti-mouse IgG (Cell Signaling, Danvers, MA, USA). Secondary antibodies used for immunofluorescence staining include: Alexa Fluor 488 goat anti-mouse (Invitrogen) and Alexa Fluor 594 goat anti-rabbit (Invitrogen).

### 4.4. Recombinant Plasmid Construction

The cDNA for full-length human Brd4 was PCR amplified and subcloned into the BamHI and NotI sites of pcDNA4/HisMax C vector (pcDNA4C, Invitrogen). For the pcDNA4C-Cdk9 construct, Cdk9 cDNA was PCR amplified and subcloned into the EcoRI and NotI sites of pcDNA4C. The E2 expression constructs pcDNA4C-E2TA, pcDNA4C-E2TR, pcDNA4C-16E2, and pcDNA4C-16E2 R37A/I73A were generated by PCR amplifying the indicated E2 gene and subcloning into the EcoRI and NotI sites of pcDNA4C. For pcDNA4C-Cdk9-E2TA, pcDNA4C-Cdk9-E2TR, pcDNA4C-Cdk9-16E2, and pcDNA4C-16E2 R37A/I73A, the Cdk9 gene was fused to the indicated E2 gene using PCR. This fusion product was then subcloned into the EcoRI and NotI sites of pcDNA4C. The constructs containing HPV16 E2 RI and/or Cdk9 D167N were generated by mutating the indicated sites using the QuikChange site-directed mutagenesis kit (Stratagene, La Jolla, CA, USA). pCMV-LacZ (β-galactosidase) was purchased from Clontech (Mountain View, CA, USA). CMV-Flag-16E2 and the plasmid encoding Brd4-CTD (pcDNA4C-NLS-Brd4-CTD were previously described [34]. The 2x2xE2BS-Luciferase reporter was described previously [13,32].

### 4.5. Luciferase Transactivation Assay

C33A cells were transfected using the calcium phosphate method and lysed in Reporter Lysis Buffer (Promega, Madison, WI, USA) at 48 h post transfection. Luciferase activities were measured according to the manufacturer’s instructions (Luciferase Assay System; Promega). In Figure 1, the luciferase activity was normalized to β-galactosidase activity measured using β-galactosidase Enzyme Assay System (Promega). For Figure 3 and Figure 4, luciferase activities calculated from three independent experiments were presented and Western blotting analyses were performed to ensure that these luciferase activities resulted from similar amount of E2 proteins.

### 4.6. Quantitative Reverse Transcription PCR (RT-qPCR)

Total RNA was isolated using a NucleoSpin RNA II Kit (Macherey-Nagel, Bethlehem, PA, USA) following the manufacturer’s instructions. Reverse transcription was performed using a 20 μL reaction mixture containing 350 ng of total RNA, oligo(dT) primer (Invitrogen), dNTP (Invitrogen), and M-MLV reverse transcriptase (Invitrogen), following the manufacturer’s instructions. Real time PCR was performed using a CFX96 real time PCR detection system (Bio-Rad, Hercules, CA, USA) with IQ SYBR Green supermix (Bio-Rad). The mRNA level of each gene was normalized to GAPDH mRNA level. Primer sequences are given in Table S1.

### 4.7. Immunoprecipitation

C33A cells stably expressing Flag-HA-tagged E2TA were treated with 500 nM JQ1(−) or JQ1(+) for 15 h. The cells were then pelleted and resuspended in buffer A (10 mM HEPES (pH 7.9), 10 mM KCl, 0.1 mM EDTA, 0.1 mM EGTA, and 1 mM dithiothreitol (DTT) supplemented with protease inhibitors (Roche)). The resuspended cells were incubated on ice for 10 min, and Nonidet P-40 was added to a final concentration of 0.6%. After vortexing and centrifugation at 3,220 x g for 5 min, the nuclear pellet was resuspended in ice-cold buffer B (20 mM HEPES (pH 7.9), 0.4 mM NaCl, 1 mM EDTA, 1 mM EGTA, and 1 mM DTT, supplemented with protease inhibitors). To extract nuclear proteins, nuclei were passed through a 21-gauge needle ten times and extracted at 4 °C for 1 h. Nuclear proteins were isolated by centrifugation at 20,817 × g for 15 min and 20 μg of protein were used as input for analysis of GAPDH protein level. The remaining lysate was diluted in buffer A and incubated with 10 μL of pre-blocked (1% BSA in PBS for 1 h at 4 °C) anti-Flag M2 affinity gel beads (Sigma) overnight at 4 °C. The beads were then washed 3 times with 150 mM KCl base buffer (20 mM Tris (pH 8.0), 10% glycerol, 5 mM MgCl_2_, 0.1% Tween-20, 150 mM KCl, and protease inhibitors (Roche)) and eluted with sample buffer. Input and IP samples were resolved on a SDS-PAGE gel and proteins were immunoblotted as described above.

### 4.8. Chromatin Immunoprecipitation (ChIP)

For ChIP analysis, formaldehyde was added directly to cell culture media to a final concentration of 1%. Fixation was completed after incubation for 10 min at room temperature and stopped by adding glycine to a final concentration of 0.125 M. Cells were scraped, collected, centrifuged and swelled in cell lysis buffer (5 mM PIPES (pH 8.0), 85 mM KCl, 1% NP-40, 0.1 mM PMSF, and 1 μg/mL leupeptin, aprotinin and Pepstatin A). After 1 h incubation on ice, nuclei were collected by centrifugation at 3,220 x g for 10 min at 4 °C, resuspended in nuclei lysis buffer (50 mM Tris-HCl (pH 8.0), 1% SDS, 10 mM EDTA, 0.1 mM PMSF, and 1 μg/mL leupeptin, aprotinin and Pepstatin A) and incubated on ice for 10 min. Samples were sonicated on ice to an average DNA length of 500 bp and centrifuged at 20,817 x g. The chromatin solution was pre-cleared with Staph A cells (pre-blocked with 1 mg/mL sheared herring sperm DNA and 1 mg/mL BSA overnight at 4 °C) for 15 min at 4 °C. Chromatin from about 10^7^ cells was incubated with 5 μg of normal rabbit IgG (NRIgG, Millipore), an affinity-purified rabbit polyclonal antibody Brd4NA (recognizes BRD4 aa 156–284) [56], or a Cdk9 antibody (Santa Cruz, sc-484). After rotating at 4 °C overnight, chromatin and antibody complexes were immunoprecipitated by mixing with pre-blocked Staph A cells at 4 °C for 15 min. Immunoprecipitates were centrifuged at 20,817 x g for 3 min. The supernatant from the NRIgG antibody sample was used as total chromatin input. Staph A immuno-complexes were washed twice with dialysis buffer (50 mM Tris-HCl (pH 8.0), 2 mM EDTA, 0.2% Sarkosyl, 0.1 mM PMSF) and four times with ChIP wash buffer (100 mM Tris, pH 9.0, 500 mM LiCl, 1% NP-40, 1% deoxycholic acid, 0.1 mM PMSF). Immuno-complexes were eluted from the Staph A cells using elution buffer (50 mM NaHCO_3_, 1% SDS). Crosslinking was reversed by adding NaCl to the eluted supernatants to a final concentration of 300 mM and incubating at 67 °C overnight. RNA was removed by incubation with RNase A at 37 °C for 30–60 min. DNA samples were purified using a PCR purification kit (Qiagen, Venlo, Netherlands) and eluted in 50 μL elution buffer. Quantitative PCR (qPCR) was performed using a CFX96 real time PCR detection system (Bio-Rad) with IQ SYBR Green supermix (Bio-Rad). Primer sequences are given in Table S1. Two micro liters of immunoprecipitated DNA or total input chromatin diluted 1:50 were used as templates for the PCR reactions.

### 4.9. Whole Genomic Extraction

Cells were treated with JQ1(−) or JQ1(+) for 15 h prior to harvest. Trypsinized cells were washed with PBS and pelleted by centrifuging at 1,811 × g for 5 min at 4 °C. The pellets were resuspended in lysis buffer (400 mM NaCl, 10 mM TrisCl (pH 7.4), 10 mM EDTA (pH 7.0), 0.2% sodium dodecyl sulfate (SDS)) and 45 μg of RNaseA (Roche) was added. The lysates were then passed through a 22-gauge needle ten times and incubated at 37 °C for 30 min. Protein was then digested with 100 μg proteinase K (Roche) overnight at 37 °C. A phenol/chloroform extraction was then performed by adding 500 μL phenol/chloroform/isoamyl alcohol (25:24:1) (Sigma) to the samples then mixing for 5 min at room temperature and microcentrifuging at 20,817 × g for 5 min. The aqueous supernatant was recovered and mixed with chloroform/isoamyl alcohol (24:1) (Sigma) for 5 min at room temperature. After microcentrifugation at 20,817 x g for 5 min, the DNA in the aqueous supernatant was isolated and precipitated by adding 2.5 volumes of isopropanol and 1/10 volume of 3 M sodium acetate and incubating overnight at −20 °C. The DNA was pelleted by microcentrifuging at 20,817 × g for 20 min at 4 °C. The pellet was washed with 70% ethanol, microcentrifuged at 20,817 × g at room temperature, dried, and resuspended in water. Viral episome levels were measured by qPCR using a CFX96 real time PCR detection system (Bio-Rad) with IQ SYBR Green supermix (Bio-Rad). Primer sequences are given in Table S1. The episomal DNA level was normalized to the GAPDH gene.

### 4.10. Immunoblotting

Cells were collected at 48 h post transfection and washed once with PBS. The cells were lysed in buffer A (10 mM HEPES (pH 7.9), 10 mM KCl, 0.1 mM EDTA, 0.1 mM EGTA, and 1 mM dithiothreitol (DTT)), supplemented with protease inhibitors (Roche)). The cells were then incubated on ice for 10 min, and Nonidet P-40 (NP-40) was added to a final concentration of 0.6%. After vortexing and centrifugation at 3,220 × g for 5 min, the nuclear pellet was resuspended in ice-cold buffer B (20 mM HEPES (pH 7.9), 0.4 M NaCl, 0.1 mM EDTA, 0.1 mM EGTA, and 1 mM DTT, supplemented with protease inhibitors), thoroughly vortexed, and mixed at 4 °C for 1 h. The nuclear proteins were isolated by centrifugation at 20,817 × g for 15 min. The samples were then resolved by sodium dodecyl sulfate-polyacrylamide gel electrophoresis (SDS-PAGE), transferred onto polyvinylidene fluoride (PVDF) membrane, and immunoblotted with specific antibodies.

### 4.11. Immunofluorescence Staining

Cells were cultured on coverslips and either immediately fixed for 20 min with 4% paraformaldehyde (PFA) in phosphate-buffered saline (PBS) or pre-extracted on ice for 10 min in CSK buffer (10 mM PIPES (pH 6.8), 30 mM sucrose, 3 mM MgCl_2_, 1 mM EGTA, 0.5% Triton X-100, from 150 to 300 mM NaCl, and supplemented with protease inhibitors) before being fixed with 4% PFA in PBS for 20 min. The coverslips were then incubated in blocking/permeabilization buffer (3% bovine serum albumin and 0.5% Triton X-100 in PBS) and then incubated with primary antibodies for 1 h at room temperature. After incubation, cells were washed 3 times using blocking/permeabilization buffer and incubated with Alexa Fluor 488 goat anti-mouse and Alexa Fluor 594 goat anti-rabbit secondary antibodies (Invitrogen) for 1 h. The cells were counterstained with DAPI.

### 4.12. Microscopy and Image Analysis

All IF images were collected using an inverted fluorescence microscope (Olympus, Tokyo, Japan, IX81) equipped with an UPlanSApo 100×/1.4 oil immersion lens (Olympus) and a high-resolution charge-coupled device camera (QImaging, Surrey, BC, Canada, FAST1394) at room temperature. Photos were taken using a 100× lens with immersion oil type-F (Olympus). Image data were analyzed and presented using SlideBook 5.0 software [60]. Images were cropped and scale bars were added using ImageJ [61]. 

## Supplementary Files

Supplementary File 1
