# Supplementary material for: The Cellular Bromodomain Protein Brd4 has Multiple Functions in E2-Mediated Papillomavirus Transcription Activation
Source: Viruses. 2014 Aug 20;6(8):3228–49. doi: 10.3390/v6083228 (PMC4147693; doi:10.3390/v6083228)
Supplement: Supplementary File 1 [file viruses-06-03228-s001.pdf]

Supplementary Materials

The Cellular Bromodomain Protein Brd4 has Multiple Functions in E2-Mediated Papillomavirus Transcription Activation

Christine M. Helfer, Junpeng Yan and Jianxin You

Figure S1. Higher E2 levels do not restore E2 transactivation function in the presence of JQ1(+). (**A**) C33A cells were cotransfected with p2x2xE2BS-Luciferase and either empty vector (Vector) or increasing amounts of E2 (1/3×, 1/2×, 1×). Empty vector was used to make the amount of DNA used for each transfection equal. These cells were treated with 500 nM JQ1(−) or JQ1(+) at 33 h post transfection and collected at 48 h post transfection for luciferase measurement. The average and standard deviation were calculated from three experiments (**B**) Cells were transfected and treated with JQ1 as in (**A**). Protein extracts were immunoblotted with anti-HPV16 E2 and anti-actin antibodies.

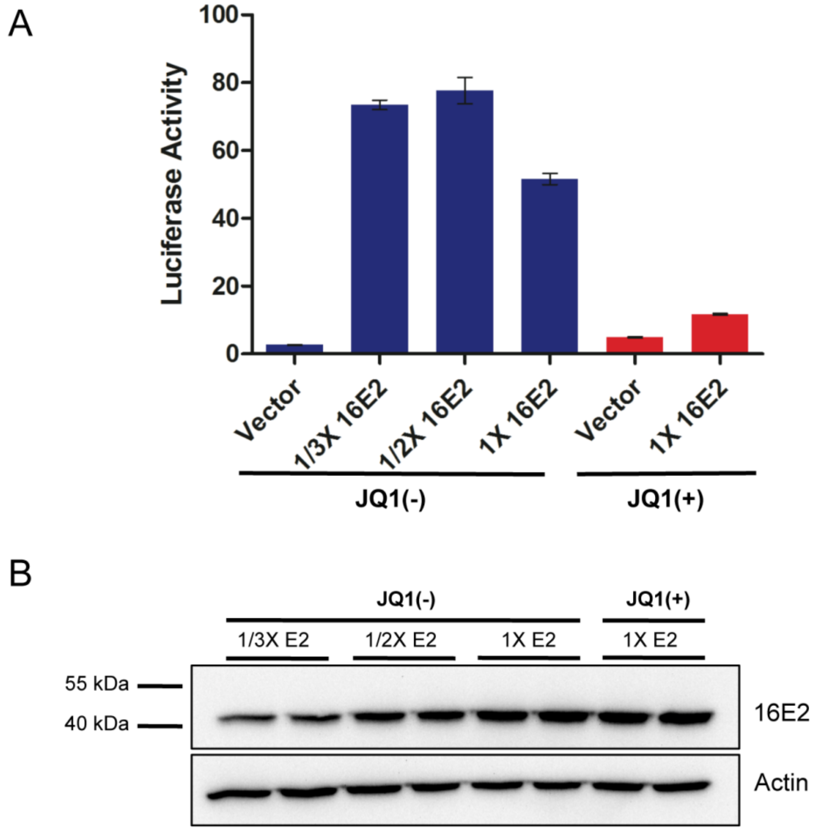

Figure S2. JQ1(+) treatment does not significantly affect papillomavirus episome level. **(A)** H2 cells were treated with 1 µM JQ1(−) or JQ1(+) for 15 h. Whole genomic DNA was extracted and the BPV1 episome level in each sample was measured using qPCR. The BPV1 episome level was normalized to the GAPDH DNA level in the samples. (**B**) W12 (clone 20863) cells were treated with 100 nM JQ1(−) or JQ1(+) for 15 h and whole genomic DNA was extracted. The HPV16 episome level in each sample was measured using qPCR. The HPV16 episome level was normalized to the GAPDH DNA level in the samples. For (**A**) and (**B**), the average and standard deviation were calculated from three independent experiments.

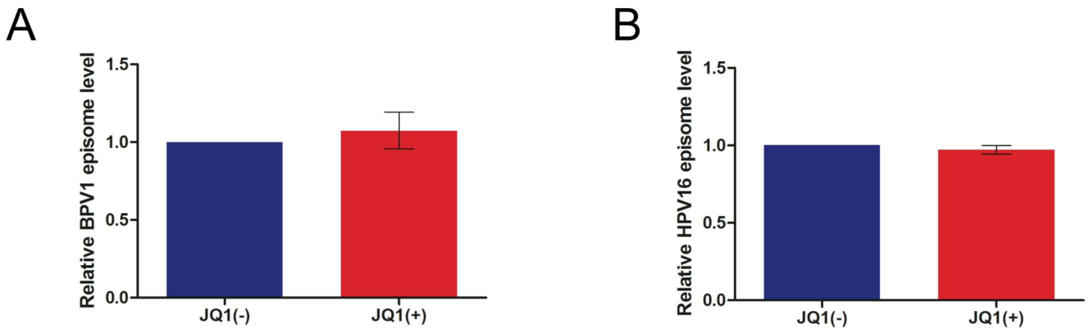

Figure S3. Raw, unmodified Western blot images used for Figure 1C. The primary antibodies used for blotting are indicated.

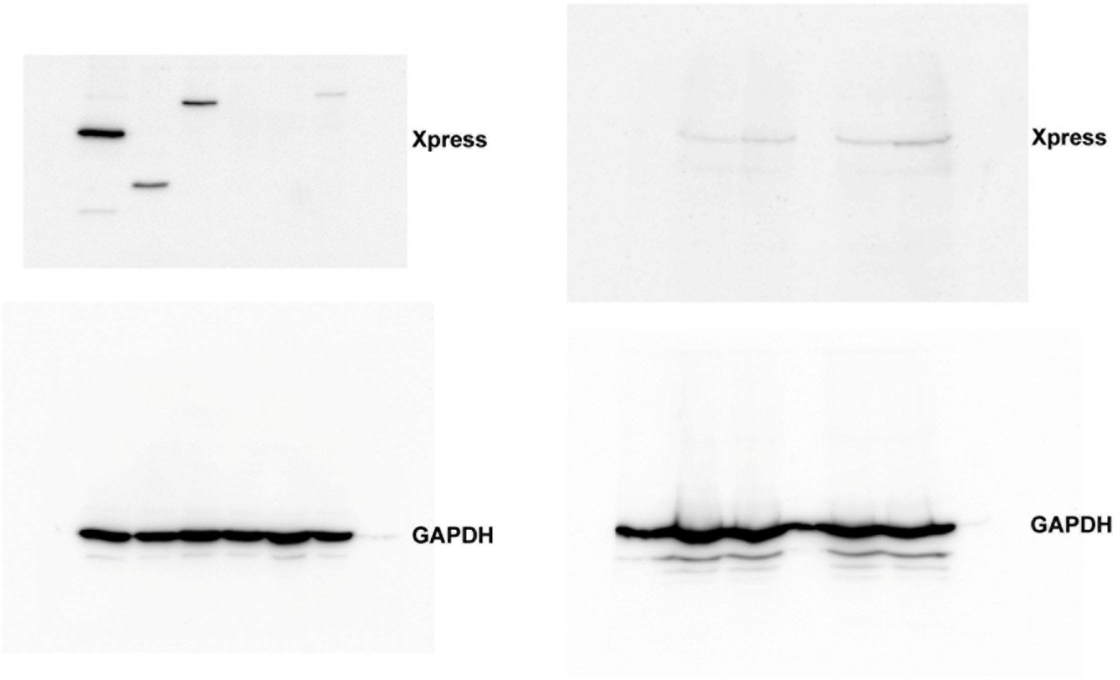

Figure S4. Raw, unmodified Western blot images used for Figure 3C. The primary antibodies used for blotting are indicated.

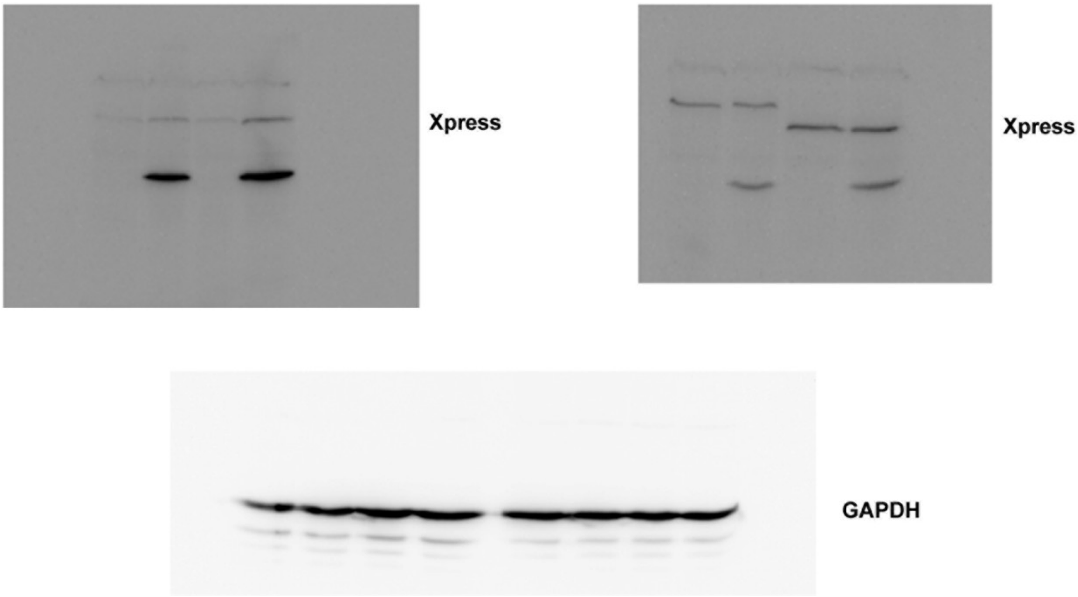

Figure S5. Raw, unmodified Western blot images used for Figure 4C. The HPV16 E2 and Actin blots are also presented in Figure S1. The primary antibodies used for blotting are indicated.

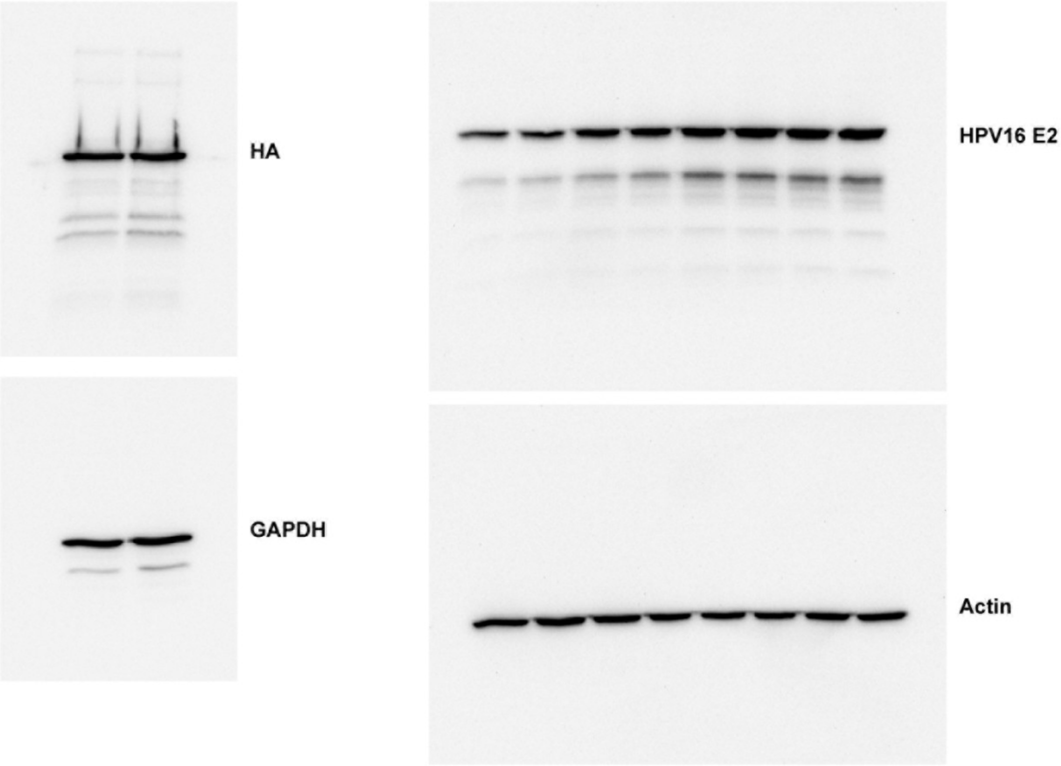

Table S1. qPCR primers. The primer sequences used in Figures 2, 5, and S2 for qPCR analysis are listed.

Primer Name	Primer Sequence
BPV1 Genome Forward	5' GAAGAGGATGGAGACAGCATGC 3'
BPV1 Genome Reverse	5' TCTGTGCGCATGTACAAATTGC 3'
HPV16 Genome Forward	5' CAATGCGACACAAACGTTCTGC 3'
HPV16 Genome Reverse	5' CTATAGAAGGATCGGAAGGG 3'
BPV1 E6 Forward	5' TCCATTCTCAGGGTTGGATTG 3'
BPV1 E6 Reverse	5' CACAGTAGCAGCATCTTATGC 3'
BPV1 E7 Forward	5' CGTTGCTGATTTTAAGTCCATGTG 3'
BPV1 E7 Reverse	5' GTCTTCACAGCAAAAGTCAGCT 3'
BPV1 E1 Forward	5' CTGACTGAGGCAGAATGTGAAAG 3'
BPV1 E1 Reverse	5' TGGAGTTTCAGATGCTTCGG 3'
BPV1 E2 Forward	5' GCTGTTAGAACTGAGAACACACTG 3'
BPV1 E2 Reverse	5' GCCTTTCTTAAAGCACCGTTTAGG 3'
Mouse GAPDH Forward	5' CCAGCCTCGTCCCGTAGA 3'
Mouse GAPDH Reverse	5' CGCCCAATACGGCCAAA 3'
HPV16 E6 Forward	5' TACCACAGTTATGCACAGAGC 3'
HPV16 E6 Reverse	5' GCTTTTCTTCAGGACACAGTG 3'
HPV16 E7 Forward	5' GCATGGAGATACACCTACATTGC 3'
HPV16 E7 Reverse	5' CGAATGTCTACGTGTGTGCTTTG 3'
HPV16 E1 Forward	5' GAAGAGGGTACGGGATGTAATG 3'
HPV16 E1 Reverse	5' CATGTGCTGTCTCTGTTTCTGC 3'
HPV16 E2 Forward	5' CAGACCTACGTGACCATATAGACT 3'
HPV16 E2 Reverse	5' CTGCACTTCCACTGTATATCCATG 3'
Human GAPDH Forward	5' GTGAAGGTCGGAGTCAACGGA 3'
Human GAPDH Reverse	5' CCATGGGTGGAATCATATTGGAAC 3'
